# Morphology of Pyramidal Neurons in the Rat Prefrontal Cortex: Lateralized Dendritic Remodeling by Chronic Stress

**DOI:** 10.1155/2007/46276

**Published:** 2007-06-11

**Authors:** Claudia Perez-Cruz, Jeanine I. H. Müller-Keuker, Urs Heilbronner, Eberhard Fuchs, Gabriele Flügge

**Affiliations:** ^1^Clinical Neurobiology Laboratory, German Primate Center, 37077 Göttingen, Germany; ^2^DFG Research Center Molecular Physiology of the Brain (CMPB), University of Göttingen, Göttingen, Germany; ^3^Department of Neurology, Medical School, University of Göttingen, 37073 Göttingen, Germany

## Abstract

The prefrontal cortex (PFC) plays an important role in the stress response. We filled pyramidal neurons in PFC layer III with neurobiotin and analyzed dendrites in rats submitted to chronic restraint stress and in controls. In the right prelimbic cortex (PL) of controls, apical and distal dendrites were longer than in the left PL. Stress reduced the total length of apical dendrites in right PL and abolished the hemispheric difference. In right infralimbic cortex (IL) of controls, proximal apical dendrites were longer than in left IL, and stress eliminated this hemispheric difference. No hemispheric difference was detected in anterior cingulate cortex (ACx) of controls, but stress reduced apical dendritic length in left ACx. These data demonstrate interhemispheric differences in the morphology of pyramidal neurons in PL and IL of control rats and selective effects of stress on the right hemisphere. In contrast, stress reduced dendritic length in the left ACx.

## 1. INTRODUCTION

The prefrontal cortex (PFC) exhibits a hemispheric specialization with respect to its functional role in the integration of affective states suggesting that the right PFC is important in eliciting stress responses (see Sullivan [1]). Uncontrollable foot shock (Carlson et al. [2]) or novelty stress (Berridge et al. [3]) resulted in a higher dopamine turnover selectively in the right PFC. The PFC has been subdivided into three main cytoarchitectonic subareas: infralimbic (IL), prelimbic (PL), and anterior cingulate cortex (ACx) (Krettek and Price [4]; Ray and Price [5]). Each of these subareas has specific cortical and subcortical connections (Vertes [6]) and distinct physiological functions. Lesion studies have shown that after acute stress, ventral (IL/PL) (Sullivan and Gratton [7]) and dorsal PFC (PL/ACx) (Diorio et al. [8]) regulate the release of corticosterone and ACTH in an opposite way. Specific behavioral responses such as diminished fear reactivity (Lacroix et al. [9]) were observed after bilateral lesions in the IL (Frysztak and Neafsey [10]), and increased fear reactivity was detected when the region PL/ACx was lesioned (Morgan and LeDoux [11]). Anxiety-like responses were observed after lidocaine infusion into the IL (Wall et al. [12]) or lesioning the right IL (Sullivan and Gratton [13]).

Recent studies in rats showed morphological changes in pyramidal neurons in the PFC following chronic restraint stress (Radley et al. [14, 15]; Cook and Wellman [16]) or after chronic corticosterone treatment (Wellman [17]). Chronic exposure to corticosterone also reduced the volume of layer II in all PFC subareas (Cerqueira et al. [18]). Chronic restraint stress for 21 days decreased the number and the length of apical dendrites in Cg1–Cg3 (corresponding to the region PL/ACx) (Cook and Wellman [16]; Radley et al. [14]), an effect accompanied by reduced spine density in the proximal portions of the apical dendrites (Radley et al. [15]). However, these studies did not investigate regional or possible hemispheric differences.

In the present study, we investigated whether pyramidal neurons in the three PFC subareas have a hemisphere-specific morphology, and whether their specific dendritic architecture would be remodeled in a lateralized manner in response to chronic stress. As reference for the exact localization of the neurons prior to their morphological reconstruction, we first identified the boundaries between the PFC subareas using antibodies against parvalbumin, the neurofilament protein SMI-32, and neuronal nuclear antigen (NeuN). To reconstruct the morphology of individual pyramidal neurons in layer III which is known to have reciprocal connections with the mediodorsal thalamic nucleus (Groenewegen [19]), we filled cells with neurobiotin using a whole-cell patch-clamp technique. Intracellular neurobiotin staining is a highly sensitive method (Pyapali et al. [20]) for visualizing neuronal processes that are not obscured by more intensely stained portions of the neurons (Hill and Oliver [21]; Oliver et al. [22]). We investigated the morphological characteristics of pyramidal cells in the three PFC subareas paying particular attention to hemispheric differences in dendritic morphology following three weeks of daily restraint stress.

## 2. MATERIALS AND METHODS

### 2.1. Animals

Adult male Sprague Dawley rats (Harlan-Winkelmann, Borchen, Germany) were housed in groups of three animals per cage with food and water *ad libitum*. Animals were maintained in temperature-controlled rooms (21 ± 1°C) with a light/dark cycle of 12 hours on, 12 hours off (lights on at 07:00). All animal experiments were performed in accordance with the European Communities Council Directive of November 24, 1986 (86/EEC), the US National Institutes of Health *Guide for the Care and Use of Laboratory Animals*, and were approved by the Government of Lower Saxony, Germany. We used the minimum number of animals required to obtain consistent data.

### 2.2. Perfusion and tissue preparation for PFC boundary identification

Male rats (*n* = 5, weighing 220–250 g) were killed by intraper-itoneal administration of an overdose of ketamine (50 mg/kg body weight; Ketavet, Pharmacia & Upjohn, Erlangen, Germany), xylazine (10 mg/kg body weight; Rompun, Bayer Leverkusen, Germany), and atropine (0.1 mg/kg body weight; WDT, Hannover, Germany). The descending aorta was clamped and the animals were transcardially perfused with cold 0.9% NaCl for five minutes, followed by cold 4% paraformaldehyde in 0.1 M phosphate buffer (PB) at pH 7.2 for 20 minutes. *Post* perfusion artifacts were prevented by postfixing heads in fresh fixative at 4°C (Cammermeyer [23]). The following day, the brains were gently removed and stored overnight in 0.1 M PB at 4°C. Brains were cryoprotected by immersion in 2% DMSO and 20% glycerol in 0.125 M phosphate-buffered saline (PBS) at 4°C.

A small hole in the left striatum was made with a thin needle to differentiate the left from the right hemisphere. The brains were then cut into blocks containing the entire PFC, frozen on dry ice, and stored at −80°C before serial cryosectioning at a section thickness of 50 *μ*m. Eight to ten complete series of coronal sections were collected and stored in 0.1 M PBS for immunocytochemistry. A stereotaxic atlas of the rat brain (Paxinos and Watson [24]) was used during the cryosectioning procedure.

### 2.3. Immunocytochemistry

Pilot experiments were performed to determine the optimal antibody concentration and incubation times. Free-floating sections were washed in 0.1 M PBS and then treated with 0.5% H_2_O_2_ for 30 minutes. After washing, nonspecific binding of antibodies was blocked by incubating the sections for one hour in 5% normal goat serum (NGS; DAKO, Glostrup, Denmark) in 0.1 M PBS containing 0.25% Triton X-100. The sections were subsequently incubated for 48 hours at 4°C with the primary antibodies, neurofilament SMI-32 (mouse-anti-SMI-32, Sigma Aldrich), parvalbumin (mouse-anti-PV; Sigma Aldrich), and neuronal-nuclei NeuN (mouse-anti-NeuN, Sigma Aldrich) at dilutions 1 : 1000, 1 : 2000, and 1 : 500, respectively, in PBS containing Triton X-100 (0.5% for SMI-32, 0.25% for parvalbumin and NeuN) and NGS (3% for SMI-32 and NeuN, 1% for parvalbumin).

Following incubation, sections were thoroughly washed with 0.1 M PBS and incubated with biotinylated goat antimouse antibody (DAKO) diluted 1 : 200 in 0.1 M PBS with 3% NGS and 0.5% Triton X-100, for 1.5 hours, followed by washing in 0.1 M PBS. The sections were then incubated with 1 : 200 horseradish peroxidase-conjugated streptavidin (DAKO) in 0.1 M PBS with 3% NGS and 0.5% Triton X-100 for 1.5 hours.

After washing, sections were stained with a DAB kit (Vector Laboratories, Burlingame, Calif, USA), which contains 3,3′-diaminobenzidine (DAB) as chromogen. Staining time in DAB was 8–10 minutes for all sections; the reaction was stopped by washing the sections in 0.1 M PBS. Sections were mounted on glass slides in 0.1% gelatin and dried overnight at 37°C, after which they were cleared in xylene for 30 minutes and finally coverslipped with Eukitt (Kindler, Freiburg, Germany). A series of adjacent coronal sections was also mounted on glass slides, dried overnight at room temperature, and stained with cresyl violet to obtain a clear comparison with the immunocytochemical images.

### 2.4. Analysis of immunocytochemically stained sections

Areal and laminar staining patterns were examined microscopically. Coronal sections were analyzed and photographed using a Zeiss Axiophot II photomicroscope (Carl Zeiss, Germany) at magnifications of 2.5 x, 10 x, and 20 x. The prefrontal cortical areas were identified and their boundaries or transition zones were outlined on photomicrographs of the sections, and a contour pattern (delineating IL, PL, and ACx subareas) was drawn and stored as a CorelDRAW file. Localization of intracellularly filled cells (see below) was then corroborated by overlapping a picture of a filled cell with a picture of a boundary contour pattern closest to the same region (anterior or posterior; see Results). SMI-32, parvalbumin, and NeuN stained sections were compared to ensure that the defined areas coincided, and were treated identically for the methods and measurements described below. Stereotaxic coordinates of the PFC were identified with the rat brain atlas (Paxinos and Watson [24]) and cortical layers in the subfields were identified using the accompanying text book (Zilles and Wree [25]).

### 2.5. Chronic restraint stress

Male Sprague Dawley rats initially weighing 150–170 g were housed in groups of three animals with *ad libitum* access to food and tap water. The first experimental phase (habituation) lasted for 14 days, during which body weight was recorded daily. Animals were randomly assigned to the experimental (*stress*) and control groups. The second phase of the experiment (restraint stress) lasted for 21 days, during which the animals of the *stress* group (*n* = 16) were submitted to daily restraint stress for six hours per day (09:00–15:00). The restraint procedure was carried out according to an established paradigm (Magariños and McEwen [26]). Briefly, rats were placed in plastic tubes in their home cages and had no access to food or water. *Control* rats (*n* = 16) were not subjected to any type of stress but were handled daily. At the end of the experiment, 24 hours after the last stress exposure, animals were weighed, deeply anesthetized with a mixture of 50 mg/mL ketamine, 10 mg/mL xylazine, and 0.1 mg/mL atropine by intraperitoneal injection, and decapitated.

Brains were rapidly removed and processed for slice preparation (see below). Increased adrenal and decreased thymus weights are indicators of sustained stress. These organs were therefore dissected immediately after decapitation and weighed. The data are expressed in milligrams per 100 grams body weight.

### 2.6. Slice preparation

After dissecting the PFC from the brain, a sagittal cut was made in the left temporal cortex with a razor blade to further differentiate the hemispheres. The blocks containing the left and the right PFC were rapidly submerged in ice-cold oxygenated artificial cerebrospinal fluid (ACSF) of the following composition (in mM): NaCl 125.0; KCl 2.5; L-ascorbic acid 1.0; MgSO_4_ 2.0; Na_2_HPO_4_ 1.25; NaHCO_3_ 26.0; D-glucose 14.0; CaCl_2_ 1.5 (all chemicals from Merck, Darmstadt, Germany). The PFC was glued to the stage of a vibratome (Vibracut 2, FTB, Bensheim, Germany) and cut in coronal, 400 *μ*m thick slices. The slices were allowed to recover for at least one hour in ACSF bubbled with 95% O_2_, 5% CO_2_ at pH 7.3, 33°C, and then kept at room temperature for up to seven hours.

### 2.7. Intracellular labeling and slice mounting

The method for intracellular labeling previously described (Kole et al. [27]) was used with some modifications. Neurobiotin was injected through borosilicate glass pipettes with 3–5 MΩ resistances, connected to an Axopatch 200B amplifier (Axon Instruments, Union City, CA, USA), using PULSE software (HEKA, Lambrecht, Germany). The standard pipette solution contained (in mM): K–MeSO_4_ 120, KCl 20, HEPES 10, EGTA 0.2, ATP (magnesium salt) 2, phosphocreatine (disodium salt) 10, GTP (Tris-salt) 0.3, and 3 mg/mL neurobiotin (Vector Laboratories).

PFC slices were transferred to a submerged recording chamber, continuously oxygenated with ACSF (flow rate: 1-2 mL/min), and maintained at 33°C. Cell bodies were visualized by infrared-differential interference contrast (IR-DIC) video microscopy using an upright microscope (Axioskop 2 FS, Carl Zeiss, Germany) equipped with a 40 x/0.80 W objective (Zeiss IR-Acroplan). Negative pressure was used to obtain tight seals (2–10 GΩ) onto identified pyramidal neurons. The membrane was disrupted with additional suction to form the whole-cell configuration. Pyramidal neurons with membrane potentials below −55 mV were excluded from the analysis. Cells were held at −70 mV for about 20 minutes.

Pyramidal cells are readily identified by their specific morphology, and only pyramidal-shaped somata located in layer III of IL, PL, and ACx (readily identified under IR-DIC video: Dodt and Zieglgänsberger [28]) were used for neurobiotin filling. Layer III pyramidal somata, visible by transillumination, tend to be smaller than layer V somata. The border between layers II and III was difficult to identify; however, cells in layer III were mainly found at a depth of about 400 *μ*m from the pial surface (Gabbott and Bacon [29]). Observation of labeled neurons in relation to the PFC boundaries (see Results) verified their location.

Neurobiotin injection lasted for about 20 minutes. Thereafter, the patch pipette was carefully withdrawn from the membrane and the slice was fixed in 0.1 M PB with 4% paraformaldehyde (pH 7.4) and stored at 4°C for at least 24 hours. Whole slices were processed free floating, first by blocking endogenous peroxidase activity in a 0.1 M PB solution containing 1% H_2_O_2_. After washing, nonspecific binding of antibodies was prevented by incubating the sections for one hour with 5% NGS (DAKO) in 0.1 M PBS and 0.3% Triton X-100. Subsequently, slices were incubated with avidin-biotin peroxidase (diluted 1 : 100; ABC, Vector Laboratories) in 1% NGS (DAKO) and 0.3% Triton X-100 overnight at 4°C. On the following day, slices were washed and left overnight in PBS. The following day, slices were equilibrated by washing them in TBS (pH 7.6) and the staining reaction was completed by incubation in a solution containing 0.04% NiCl_2_, 0.5 mg/mL DAB, and 0.01% H_2_O_2_ (Vector Laboratories) in TBS until a dark brown color appeared, typically in less than 10 minutes. The reaction was terminated by several washes in fresh 0.1 M PBS and finally in double-distilled water. Tissue sections were then dehydrated in an ascending series of ethanols (30%–100%), cleared with two 10-minute incubations in xylene and flat-embedded in Eukitt (Kindler) on glass slides. Slices from at least one stressed and one control animals were always processed simultaneously.

### 2.8. Neuronal reconstruction and morphometric analysis

Labeled cells were examined by light microscopy to ensure that they fulfilled the following criteria for the three-dimensional reconstruction: (1) a clearly visible and completely stained apical dendritic tree; (2) at least three main basilar dendritic branches, each branching at least to the third-degree branch order; (3) soma location in layer III of an identified PFC subarea; and (4) visibility of the most distal apical dendrites with dense labeling of the processes (Kole et al. [27]; Radley et al. [15]). To ensure that the analysis was performed blind, each slide was coded by an independent observer prior to neuronal reconstruction, and the code was not broken until all analyses were completed. In a few cases, cell coupling was observed (<1%); such cells were omitted from the analysis, because the dendrites could not be assigned unequivocally to a single neuron. Somata of intracellularly labeled cells were located at 60–70 *μ*m depth from the slice surface allowing reconstruction of almost all their main dendritic branches. Compromised cells that had truncated main apical or first-order basilar branches were omitted from the analysis. In each animal, 12 neurons were filled with neurobiotin, six in the left and six in the right hemisphere, randomly distributed among the three areas of interest. Complete and optimally labeled pyramidal neurons meeting the above criteria were reconstructed and morphological parameters were quantified using NeuroLucida software (MicroBrightField, Inc., Colchester, Vt, USA) in combination with an automated stage and focus control connected to the microscope (Zeiss III RS). Data were collected as line drawings consisting of *X*, *Y*, and *Z* coordinates. Dendritic length was measured by tracing dendrites using a 40 x (N.A. 0.75) objective, giving a final magnification of 40 000 x on the monitor. The step size of the circular cursor was 0.16 *μ*m, sufficiently below the limits of light microscopy resolution (about 0.25 *μ*m). Numerical analysis and graphical processing of the neurons were performed with NeuroExplorer (MicroBrightField). Sholl plots (Sholl [30]) were constructed by plotting the dendritic length as function of distance (corresponding to the radius) from the soma center, which was automatically set to zero. The length of the dendrites within each subsequent radial bin at 10 *μ*m increments was summed.

Ethanol dehydration and xylene clearance are known to cause tissue shrinkage (Pyapali et al. [20]). However, previous analyses from our laboratory suggested that the linear shrinkage correction has no direct effect on data used for morphological comparative analysis (Kole et al. [27]). Therefore, we did not apply any correction factor.

### 2.9. Statistical analysis

Body weight (BW) and relative organ weight (in milligrams per 100 grams of BW) of control and stress animals at the end of the experiment were compared using the unpaired *t*-test.

The total number of labeled neurons that fulfilled the above criteria to be analyzed was 69 in the control and 70 in the stress group. Since these labeled cells were not evenly distributed among the animals, we calculated the means of the morphometric data for each hemisphere/animal. These mean data served as analysis unit for the statistical evaluation and are indicated as “*n*” in the tables. Data for the total length of dendrites, the total number of branching points, and the total number of branches were evaluated by two-way ANOVA (factors: hemisphere × group) (Statistica software package, Release 6.0 StatSoft Inc., Tulsa, Okla, USA). Numbers of branches per branch order were evaluated using three-way ANOVA (factors: branch order × hemisphere × group). Sholl analysis data were evaluated with three-way repeated measures ANOVA (factors: hemisphere × group × radius) (SPSS version 12.0, SPSS Inc., Chicago, Ill, USA). Bonferroni's post hoc test was used in all cases. Because the morphology of the pyramidal cells shows complex differences along the dendritic trees we restricted our post hoc analyses to distinct radii (10 *μ*m, 20 *μ*m, 30 *μ*m, etc.) and single branch orders (1st, 2nd, 3rd order, etc.). Data are presented as mean ± SEM (standard error of the mean). Differences were considered statistically significant at *P* < .05.

## 3. RESULTS

### 3.1. Prefrontal cortex boundaries definition

According to previous descriptions, the rat PFC can be divided into three subareas: IL, PL, and ACx. As a basis for the reliable localization of neurobiotin labeled pyramidal neurons in the present study, we visualized the boundaries of these subareas using specific antibodies. The three subareas that were reliably found at the same location in all investigated brains were defined as showing differential staining patterns with at least two staining methods.

**Figure 1:**
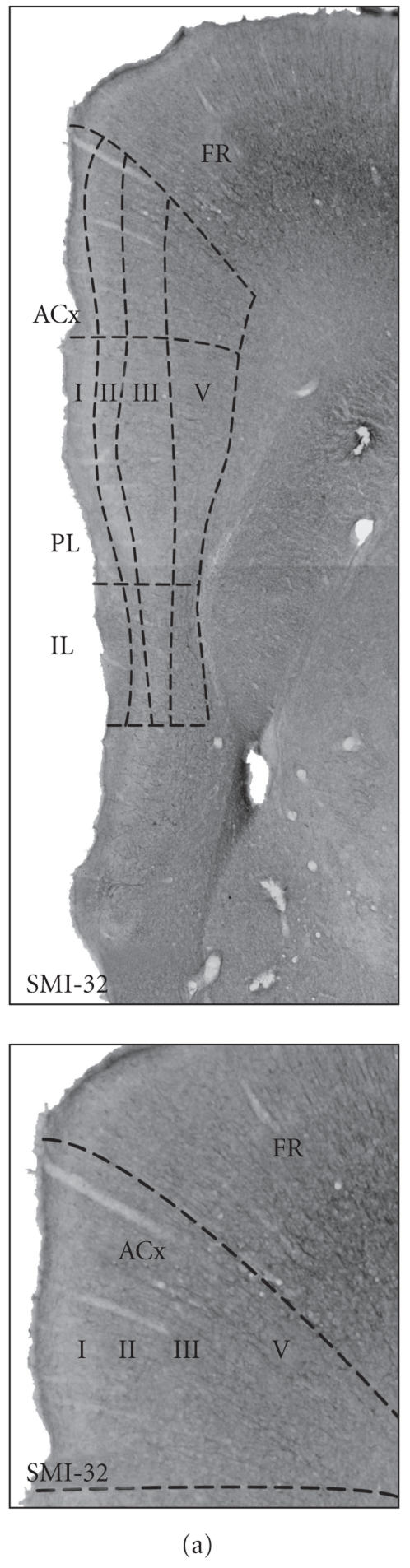

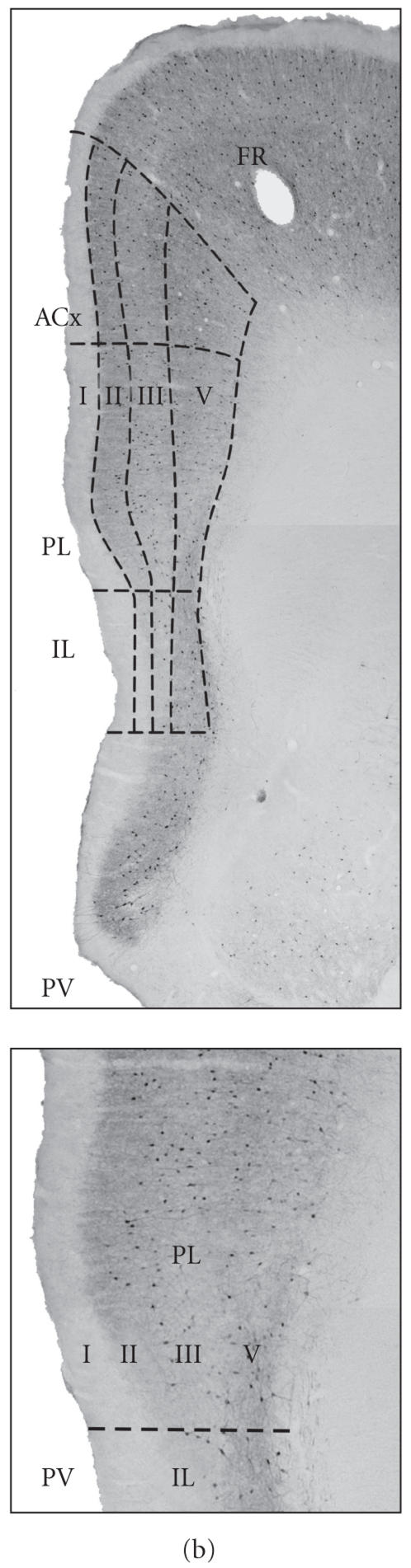

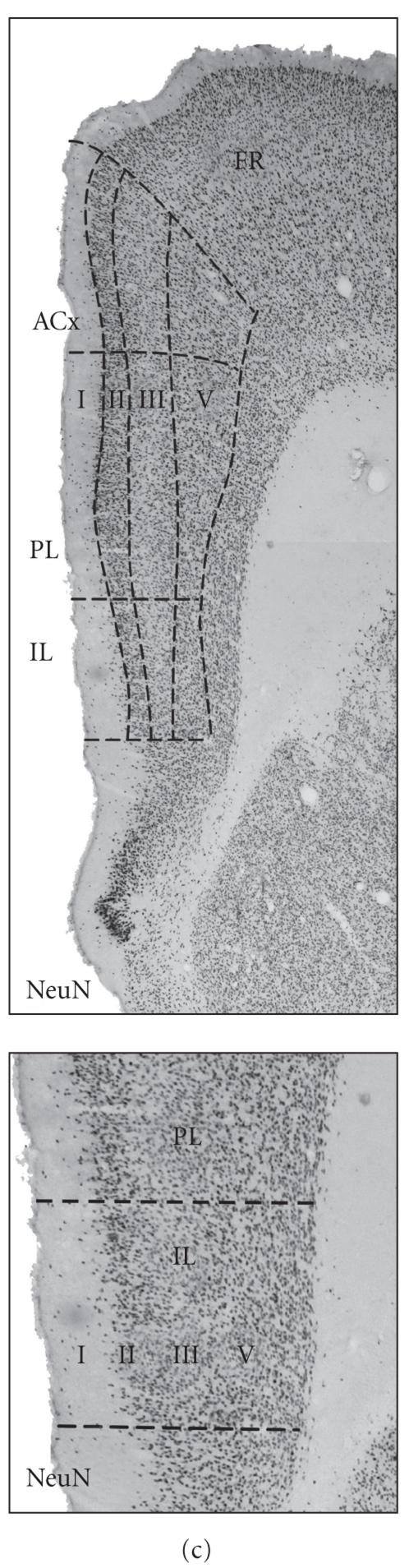

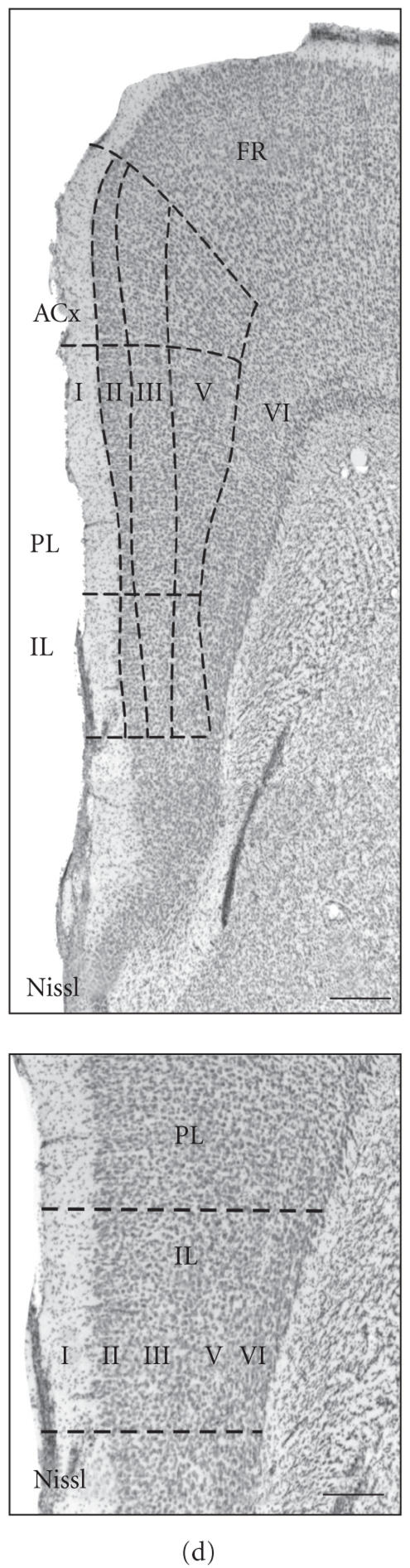
Boundaries of the PFC subareas in the rat visualized with antibodies. In the anterior PFC (3.70 − 2.20 mm from bregma), three subareas can be distinguished: IL, PL, and ACx. Pictures in the lower panels show the same sections at higher magnification. (a) Staining with the SMI-32 antibody shows the border between PL and ACx, and between ACx and premotor cortex (FR). (b) Parvalbumin (PV) is a good marker to distinguish IL from PL. The PV antibody stains neurons in layer V and in the other cortical layers in all three subareas. (c) The NeuN antibody strongly labels layer II, and also layers I–V can be easily distinguished with this antibody. (d) With Nissl staining, it is possible to distinguish layer I but not the other cortical layers. Scale bars: 500 *μ*m.

Immunocytochemical staining with SMI-32 antibody gave a staining pattern that differentiates PL from ACx, and ACx from the premotor cortex in dorsal regions of the PFC (Figure 1(a)). In the PL, the SMI-32 antibody labeled layers III and V. This pattern became lighter and narrower in the ACx, where layer III was lightly stained whereas layer V was darker and broader. ACx could be distinguished from the premotor cortex because in the latter, the deep layers were intensely labeled by the SMI-32 antibody (Figure 1(a), lower panel).

Parvalbumin proved to be a good marker to distinguish all PFC subareas and their respective layers. In IL, layer II was only lightly stained, layer III was slightly darker and layer V showed a pronounced staining. In the PL, layer II was distinctly stained by the PV antibody and layer III appeared wider than in the IL. The strong staining of layer V observed in the IL gradually disappeared in the PL. In the ACx, all layers had more parvalbumin-immunoreactive cells compared to PL. Layer II in ACx showed darker staining compared to the PL (Figure 1(b)).

Immunoreactivity for NeuN provided a boundary between IL and PL, and a clearly layered pattern in all PFC subareas with pronounced staining of layer II (Figure 1(c)). The IL was distinguished by a wide layer I and by densely packed cells in layers II. Compared to IL, the PL had a lighter layer III, and a broader layer V. In ACx, layer V was again broader than in the PL (Figure 1(c)). Using Nissl dyes, layer I can be clearly distinguished, however, it is difficult to distinguish the other cortical layers and to detect borders between PFC subareas (Figure 1(d)).

By comparing the location of each neurobiotin filled layer III neuron (see below) with the boundary patterns described above, we were able to accurately define its subarea-specific location.

### 3.2. Intracellular labeling with neurobiotin and dendritic reconstruction

**Figure 2:**
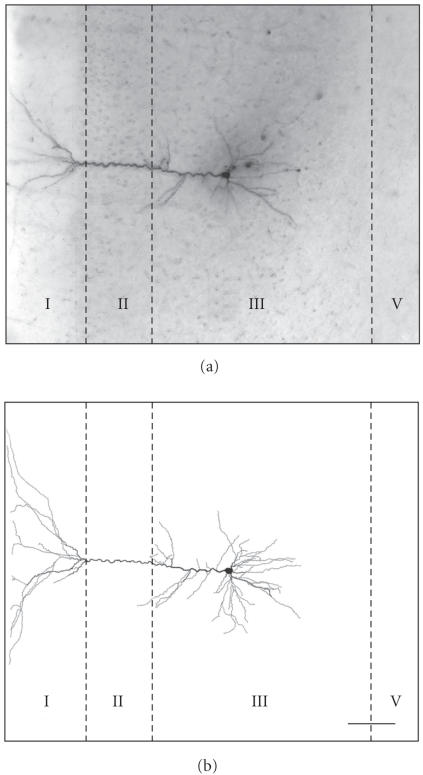
Example of an intracellularly labeled and reconstructed pyramidal neuron in the PFC of a control rat. (a) Photomicrograph of an intracellularly labeled pyramidal neuron in layer III of the prelimbic subarea (left hemisphere). (b) Line drawing of the neuron shown in (a) (reconstruction with NeuroLucida). The relative position of the pyramidal cell is shown by lines indicating the cortical layers (I–V). Scale bar: 100 *μ*m.

Intracellular neurobiotin labeling provides a reliable and sensitive method to study dendritic morphology (Paypali et al. [20]). In all experimental groups, there was complete staining of the dendritic branches with distal dendrites being reliably visualized (Figure 2). The fact that during injection of the dye cells were alive and healthy and the use of relatively thick slices (400 *μ*m) increased the probability to reconstruct complete dendritic arbors without compromised branches. In both groups, *control* and *stress*, we filled a total of 384 cells of which 36% (139 cells) fulfilled the criteria for complete staining suitable for a quantitative analysis of dendritic morphology. Since these labeled cells were not evenly distributed among the animals and to avoid any bias we calculated means/hemisphere/animal. These means served as analysis units for the statistical evaluation (see below).

### 3.3. Hemispheric differences in dendritic morphology

*Sholl* *analysis (left versus right)*

**Figure 3:**
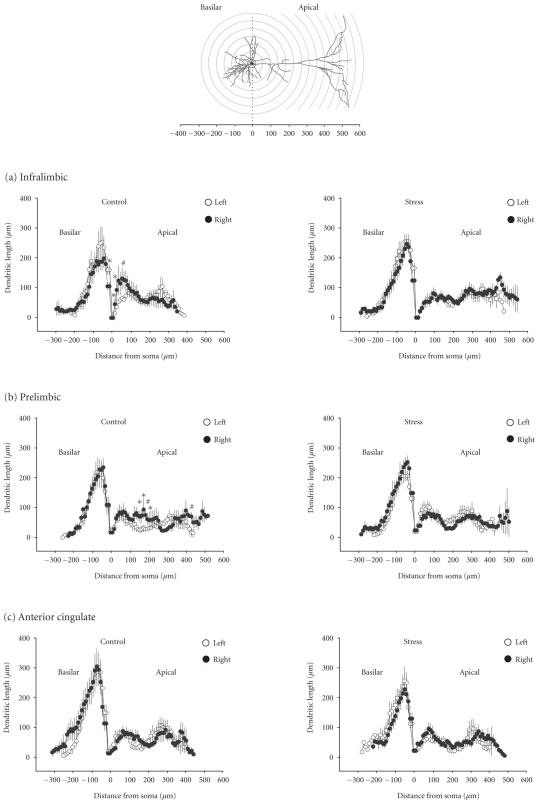
Sholl analysis of dendrites on pyramidal cells in the left (open circles) and the right hemisphere (closed circles) of controls (left panel) and stressed rats (right panel). Basilar dendrites are plotted to the left and apical dendrites to the right as a function of the distance from the soma center (0). The schematic drawing in the top panel illustrates the Sholl circles that correspond to the distances from soma depicted in (a) infralimbic, (b) prelimbic, and (c) anterior cingulate. Data points represent the sum of all dendrites detected at the respective distance (radius) from the soma center set as zero (mean ± SEM). Symbols indicate significant differences within each 10 *μ*m ring determined by ANOVA with Bonferroni's post hoc test (∗*P* < .05, #*P* < .01).

For a close inspection of the dendritic trees in the left and the right hemisphere, Sholl analyses were performed (Figure 3).

For the basilar dendrites in the IL, three-way ANOVA performed with data from both groups, control and stress, (factors: hemisphere × group × radius) revealed significant effects of hemisphere (F_(1,572)_ = 6.12, *P* < .05) and radius (F_(29,572)_ = 22.55, *P* < .001). Bonferroni's post hoc test indicated a significant interhemispheric difference for basilar dendrites in controls at 10 *μ*m from soma (df = 31, *P* < .05) (Figure 3(a), left panel). Also for apical dendrites in the IL three-way ANOVA revealed a significant effect of hemisphere (F_(1,1086)_ = 24.18, *P* < .001) but no reliable effect of radius. Bonferroni's post hoc test showed that in the control animals, apical dendrites in the right hemisphere were longer than in the left hemisphere. The sites where these right-left differences occurred were proximal to the soma, at 10, 20, and 60 *μ*m (df = 30, *P* < .05 in all cases) (Figure 3(a), left panel).

In the PL, Sholl analysis of the basilar dendrites displayed significant effects of hemisphere (F_(1,780)_ = 11.91, *P* ≤ .001) and radius (F_(29,780)_ = 43.81, *P* < .001) (Figure 3(b)). However, the post hoc test depicted no reliable difference between basilar dendrites in the left and the right hemisphere of controls. Apical dendrites in the PL also showed a positive effect of hemisphere (F_(1,1018)_ = 4.07, *P* < .05) and a weak effect of radius (F_(59,1018)_ = 1.42, *P* < .05). Bonferroni's post hoc test revealed significant interhemispheric differences in middle and distal portions of the apical dendritic tree of controls (at 180 and 420 *μ*m from soma, df = 25, *P* < .01; at 160, 170, and 190, df = 25, *P* < .01) (Figure 3(b), left panel).

For the basilar dendrites in the ACx, three-way ANOVA indicated no reliable interhemispheric difference but only an effect of radius (F_(29,399)_ = 15.55, *P* < .001). For the apical dendrites in ACx, ANOVA revealed a positive effect of the hemisphere (F_(1,841)_ = 7.81, *P* < .01) and an effect of radius (F_(51,841)_ = 3.11, *P* < .001). However, the post hoc test showed no interhemispheric difference with respect to apical dendrites in the ACx of controls (Figure 3(c), left panel).

These data demonstrate a lateralized morphology of apical dendrites on pyramidal neurons in IL and PL but not in the ACx of control rats.

*Total dendritic length (left versus right)*

The total length of basilar and apical dendrites in control rats showed no significant interhemispheric differences although apical dendrites in the right tended to be longer than in the left hemisphere (Table 1).

*Dendritic* *branches (left versus right)*

The complexity of the apical and basilar dendritic trees in the two hemispheres was determined by analyzing numbers of branching points and branches (Table 2).

For basilar dendrites, two-way ANOVA indicated no reliable interhemispheric differences for the total number of branching points and branches in any of the subareas (Table 2). Numbers of branches of distinct branch orders were evaluated by three-way ANOVA (factors: hemisphere × group × branch order).

For basilar dendrites in the IL, an effect of the hemisphere (F_(1,182)_ = 10.48, *P* < .05) and of the branch order (F_(6,182)_ = 60.82, < .001) was found (details not shown). For IL apical dendrites, numbers of branches of distinct branch orders revealed an effect of hemisphere (F_(1,288)_ = 4.73, *P* < .05) and of branch order (F_(11,288)_ = 13.74, *P* < .001) (Table 2). Bonferroni's post hoc test showed reliable interhemispheric differences for the number of branches of the orders 4, 6, and 11 (df = 24, *P* < .05 in all cases) and of branch order 12 (df = 24, *P* < .01). In the left IL, dendritic branches of the orders 11 and 12 could not be observed, but were only present in the right IL (Table 2).

For basilar dendrites in the PL, three-way ANOVA depicted no effect of the hemisphere but only an effect of the branch order (F_(6,168)_ = 68.23, *P* < .001). Also for apical dendrites in the PL there was no effect of the hemisphere but only an effect of the branch order (F_(10,253)_ = 9.86, *P* < .001). The post hoc test showed no significant interhemispheric difference for any branch order in the PL (Table 2).

In the ACx of control rats, there were no significant interhemispheric differences with respect to the total number of apical and basilar branches and branching points. Three-way ANOVA depicted only effects of the branch order (basal: F_(5,118)_ = 36.11, *P* < .001; apical: F_(10,220)_ = 9.92, *P* < .001) (Table 2).

### 3.4. Stress effects on dendritic morphology

*Total* *dendritic length and Sholl analysis (stress effects)*

Stress reduced the total length of apical dendrites on pyramidal neurons in the PL selectively in the right hemisphere (Table 1). No other stress effect on the total length of dendrites was observed.

**Figure 4:**
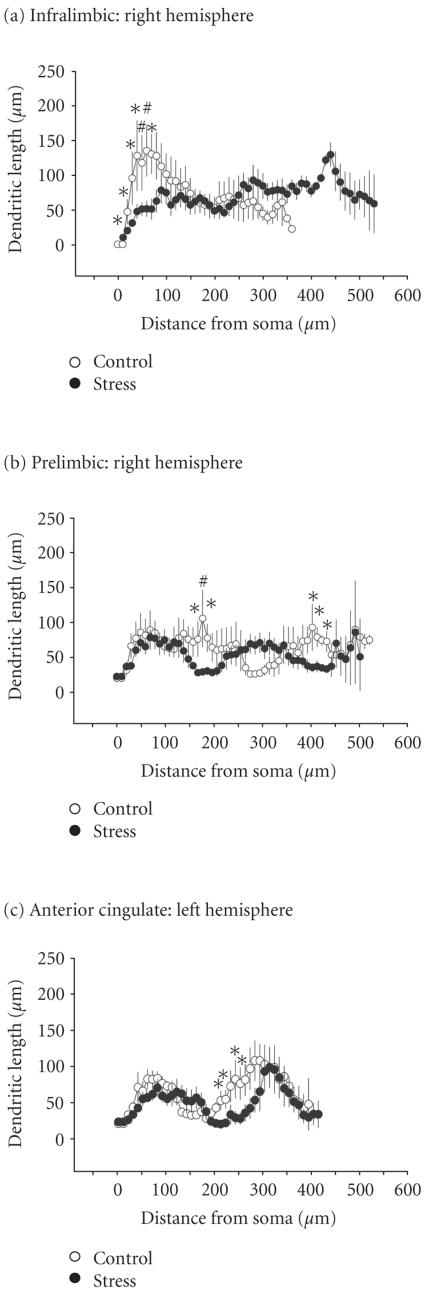
Comparisons of apical dendrites in the right hemispheres of IL and PL, and in the left ACx of stressed (closed circles) and control rats (open circles). Apical dendrites were plotted as a function of distance from the soma center (0). (a) and (b) show the effects of stress in the right hemisphere of the infralimbic and prelimbic, respectively. (c) show the effects of stress on the left hemisphere of the anterior cingulate. Data points represent the sum of all dendrites detected at the respective distance (radius) from the soma (mean ± SEM). Symbols indicate significant differences detected within each 10 *μ*m ring as determined by three-way repeated measures ANOVA (∗*P* < .05, #*P* < .01).

Dendrites of pyramidal neurons in stressed rats are shown in Figure 3 (right panel) and in Figure 4. For basilar dendrites in the IL, three-way ANOVA depicted no reliable effect of stress. These dendrites also displayed no significant left-right difference in stressed animals (Figure 3(a), right panel).

For apical dendrites in the IL, three-way ANOVA revealed an interaction hemisphere × group (F_(1,1086)_ = 5.43, *P* < .05), but the post hoc test depicted no significant left-right difference for apical dendrites at defined distances from the soma (Figure 3(a), right panel). However, in the right IL of stressed animals, the length of proximal dendrites at several sites was significantly shorter than in controls (at 10, 20, 30, 40, and 70 *μ*m, df = 30, *P* < .05; at 50 and 60 *μ*m, df = 30, *P* < .01) (Figure 4(a)).

In the PL of stressed rats, no reliable interhemispheric differences with respect to apical dendrites on pyramidal neurons were observed (Figure 3(b), right panel). Three-way ANOVA for apical branches in the PL, performed with data from all groups, showed an interaction hemisphere × group (F_(1,1018)_ = 17.40, *P* < .001). The post hoc test indicated that in the right PL, stress reduced the length of apical dendrites at 160, 170, 190, 420 *μ*m (df = 25, *P* < .05) and at 180 *μ*m (df = 25, *P* < .01) (Figure 4(b)).

For basilar dendrites in the ACx, three-way ANOVA depicted an effect of group (F_(1,399)_ = 9.23, *P* < .01) and an interaction hemisphere × group (F_(1,399)_ = 6.40, *P* < .05). Also for the apical dendrites, three-way ANOVA showed an effect of group (F_(1,841)_ = 9.34, *P* < .01) but no interaction. In the left hemisphere of the ACx, stress reduced the length of apical dendrites at certain distances from soma (210, 220, 240, and 250 *μ*m; df = 21, *P* < .05 in all cases) (Figure 4(c)). Also for the right ACx, the post hoc test depicted a significant stress effect (at 20 *μ*m from soma; df = 21, *P* < .05).

*Dendritic* *branches (stress effects)*

The effect of stress on the total number of branching points and the total number of dendritic branches was also analyzed (Table 2).

For apical dendrites in the IL, both basilar and apical dendrites showed no effect of group and no interaction. However, the post hoc test depicted a significant stress effect on numbers of branches of the order 12 in the right IL (df = 24, *P* < .01). These dendritic branches could not be observed in stressed animals (Table 2).

In the PL, the branching pattern of basilar dendrites was apparently not affected by stress. For the total number of apical branching points, two-way ANOVA revealed an interaction group × hemisphere (F_(1,31)_ = 3.28, *P* < .05) represented by a reliable stress induced decrease in the total number of branching points in the right hemisphere (df = 29, *P* < .05). Stress also reduced the number of branches of the order 3 selectively in the right hemisphere of the PL (interaction: group × hemisphere; F_(1,253)_ = 7.61, *P* < .01; post hoc test: df = 23, *P* < .05) (Table 2).

For basilar dendrites in the ACx, three-way ANOVA showed an interaction hemisphere × group (F_(1,118)_ = 5.32, *P* < .05). For apical dendrites, there was a reliable effect of group (F_(1,220)_ = 5.46, *P* < .05) represented by a stress induced increase in the number of branches of the order 3 in the right hemisphere (df = 20, *P* < .05). Moreover, there was a significant left-right difference with respect to order 3 branches in the ACx; no dendritic branches of this order could be observed in stressed rats (df = 20, *P* < .05) (Table 2).

These data show that chronic restraints stress affects dendrites in the right hemisphere of IL and PL. In contrast in the ACx, it is the left hemisphere that is affected by stress.

### 3.5. Effects of chronic restraint stress on body and organ weights

To assess the physiological effects of chronic stress, we measured body weight (BW) and weights of thymus and adrenal glands. In rats subjected to the restraint stress for 21 days, body weight at the end of the experiment was significantly lower than in controls (*control*: 328.9 ± 8.8 g; *stress*: 292.3 ± 7.0 g; *t* = 3.205, *P* < .05). Adrenal weight was significantly increased in stressed animals (*controls*: 13.66 ± 0.36 mg/100 g BW; *stress*: 16.01 ± 0.86 mg/100 g BW; *t* = 2.452, *P* < .05) and thymus weight was significantly reduced (*controls*: 120.10 ± 5.84 mg/100 g BW; *stress* 100.40 ± 4.27 mg/100 g BW; *t* = 2.755, *P*< .05). These results agree with previous reports on physiological changes induced by chronic restraint stress (Magariños and McEwen [26]; Watanabe et al. [31]; Wellman [17]).

## 4. DISCUSSION

In the first part of this study, we identified the boundaries between the three PFC subareas. The border between PL and ACx could be visualized with the SMI-32 antibody which labels neurofilaments (Sternberger and Sternberger [32]). The parvalbumin antibody, which stains a subpopulation of cortical interneurons (Gabott and Bacon [29]), strongly stained layer V and was suitable for recognizing the boundaries between IL and PL. The antibody against NeuN, a selective marker for neurons (Mullen et al. [33]), proved to be better than conventional Nissl staining at defining cortical layers II and III. Delineation of the subarea boundaries and of cortical layers was a prerequisite for the exact localization of pyramidal neurons within the rat PFC.

Because projections from the mediodorsal thalamic nucleus principally target layer III and V of the PFC (Uylings et al. [34] Gabbott et al. [35]; Krettek and Price [4]) we analyzed pyramidal cells exclusively in layer III. The neurons that we investigated showed apical dendrites extending to the brain surface, and the distance of their somata was up to 550 *μ*m from the pia mater. Therefore, their location agreed with a previous description of the PFC layers (Gabbott and Bacon [29]). In contrast, Brown et al. [36] reported that the somata of the pyramidal neurons they examined were located closer to the cortical surface (distance of 250–280 *μ*m), probably in layer II. It is important to mention that intracellular neurobiotin labeling allows a better staining of distal dendritic branches compared to conventional methods such as Golgi staining (Pyapali et al. [20]).

### 4.1. Lateralized pyramidal neuron morphology in the PFC of control rats

We found intrinsic morphological asymmetries in the pref-ronto-cortical pyramidal cells of control animals. In PL and IL, there were interhemispheric differences in the length of apical dendrites at certain distances from the soma. These are new findings, because previous studies addressing the morphology of pyramidal neurons in the PFC did not discriminate between the hemispheres (Wellman [17]; Cook and Wellman [16]; Radley et al. [14]). Intrinsic asymmetries have been observed before in several regions of the human cerebral cortex, for example, in entorhinal, auditory- and language-associated cortices (Hayes and Lewis [37]; Hutsler [38]). Simic et al. [39] found larger pyramidal neurons in the human left entorhinal cortex compared to the right, and hypothesized that this asymmetry reflects a more extended dendritic arborization and enlarged neuropil volume in the left hemisphere. The present data show that in PFC subareas PL and IL of the rat, the length of apical dendrites at certain distances from soma differs between the hemispheres.

### 4.2. Hemispheric remodeling of dendrites by chronic stress

In PL and IL, chronic stress abolished the interhemispheric differences in the length of dendrites observed at certain distances from the soma. In the right PL, stress also reduced the total length of the apical dendrites. The stress-induced factors that lead to these changes are not yet known, however, one may speculate that dopamine which is known to decrease excitability of PFC pyramidal neurons plays a role (Jedema and Moghaddam [40]; Gulledge and Jaffe [41]). Electrophysiological recordings have shown that dopamine enhances the spatiotemporal spread of activity in the rat PFC, at least in part via layer III pyramidal neurons (Bandyopadhyay et al. [42]). As mentioned above, stress can increase dopamine turnover in the right PFC (Carlson et al. [2]; Berridge et al. [3]; Slopsema et al. [43]; Thiel and Schwarting [44]) and chronic stress reduces the spontaneous activity of dopaminergic neurons in the ventral tegmental area (VTA) which project to the PFC (Moore et al. [45]; Benes et al. [46]). In coincidence with this, it was found that repeated stress reduces dopamine (Mizoguchi et al. [47]), norepinephrine (Kitayama et al. [48]) and serotonin in the PFC (Mangiavacchi et al. [49]). Although it is not known whether in the present study, the chronic restraint stress induced a deficit in dopamine and/or other monoamines, it may be hypothesized that the changes in dendrites observed here are related to maladaptive neurochemical processes.

In contrast to PL and IL, pyramidal neuron dendrites in the ACx of control rats showed no interhemispheric differences, but the stress induced a left-right difference. Previous reports described a stress induced decrease in apical dendritic length using a similar (Cook and Wellman [16]; Brown et al. [36]) or the same stress protocol (Radley et al. [14]). While we investigated only layer III pyramidal neurons, the former studies focused on cells more widely distributed over layers II-III (Cook and Wellman [16]; Radley et al. [14]). Dendritic architecture is crucial for connectivity within neuronal networks. Sensory input or environmental enrichment has been shown to promote the formation of spines on proximal dendrites (Turner and Lewis [50]). In hippocampal pyramidal neurons, extensive dendritic sprouting and enhanced spine density were observed when axonal afferents were increased (Kossel et al. [51]) whereas the loss of afferents can lead to dendritic atrophy (Valverde [52]; Benes et al. [53]; Deitch and Rubel [54]). One may speculate that the stress-induced dendritic changes observed in the present study are related to alteration in axonal input. The observation that the dendritic alterations emerged at certain distances from soma may be related to the fact that axonal input to the PFC is site-specific and depends on the cortical layer.

### 4.3. Potential consequences of dendritic remodeling and clinical implication

The stress-induced reduction in the total length of dendrites in the right PL is in line with previous findings showing lateralization of the PFC mediated stress response. Right side lesions of the IL/PL reduced the peak stress-induced corticosterone response (Sullivan and Gratton [7]) and decreased anxiety (Sullivan and Grattron [13]), suggesting that a compromised right PFC activity results in a lack of control over the physiological and behavioral responses to stress. Especially the prelimbic and the anterior cingulate cortex are important to react to environmental stimuli (Cardinal et al. [55]). Therefore, one may assume that alterations in the morphology of PFC pyramidal neurons have an impact on the stress response.

Similar stress-related processes as in the PFC have been observed in the amygdala. Chronic restraint stress *increased* the length of apical dendrites of pyramidal cells in the basolateral amygdala (Vyas et al. [56]) which sends projections to and receives input from PL and ACx (Vertes [6]). Like in the PFC, the activity of the basolateral amygdala appears to be lateralized under stress conditions (Adamec et al. [57]). Under normal conditions, the PFC inhibits the basolateral amygdala (Rosenkranz and Grace [58]); but under stress, this inhibition might be impaired thus contributing to an over-reactivity of this nucleus. It is possible that the morphological remodeling of the pyramidal neurons in the rat PFC that we describe in the present study is related to a presumptive stress-induced change in information transfer between PFC and amygdala.

Lateralization appears to be characteristic for normal PFC functioning. Studies in humans indicated that reduced lateralization correlates with pathological conditions or with aging processes as fronto-cortical activity during cognitive performance tends to be less lateralized in old compared to young adults (Dolcos et al. [59]). A recent investigation on the human dorsolateral PFC demonstrated a hemispheric asymmetry in pyramidal cell density in normal subjects (higher density left compared to right), which was reversed in schizophrenic patients (Cullen et al. [60]). Rotenberg [61] suggested that in depressed patients, the right PFC hemisphere is over-activated, and subjects with major depression displayed a reduced size of neurons in layer III of the right orbitofrontal cortex (Cotter et al. [62]). However, studies in depressed patients that did not focus on hemispheric differences reported on decreased activity in the PFC area ventral to the genu of the corpus callosum (Drevets et al. [63]), and on reduced cortical thickness, glial size, and glial densities in supragranular layers of the orbitofrontal cortex (Rajkowska et al. [64]). Our study in rats shows that chronic restraint stress has a strong effect on the morphology of pyramidal neurons in the right hemisphere, at least in PL and IL.

The neurophysiologcal consequences of the dendritic alterations are not yet known. A shortening of even a few dendrites on CA1 hippocampal pyramidal neurons enhanced the back-propagation of action potentials (Golding et al. [65]; Schaefer et al. [66]). Moreover, experiments in our laboratory revealed that a stress-induced decrease in the length of apical dendrites of CA3 pyramidal neurons in the rat hippocampus correlates with reduced onset latency of excitatory postsynaptic potentials (Kole et al. [27]). However, functional studies are required to assess the physiological implications of such morphological remodeling in the rat PFC.

## 5. CONCLUSIONS

This is the first study showing intrinsic hemispheric differences in the dendritic morphology of pyramidal neurons in subareas of the rat PFC. In PL and IL of control rats, interhemispheric differences in the length of apical dendrites at certain distances from the soma were observed. Chronic stress abolished these right-left differences and reduced the total length of apical dendrites in the right PL. In contrast in the ACx, there was no hemispheric difference in controls but stress induced a left-right difference. These chronic stress-induced regional changes may be correlated with the specialized functions of PFC subareas in stress-related pathologies, and provide additional support for previous studies of stress-dependent activation of the right PFC.

## Figures and Tables

**Table 1:** Total length (sum of all dendrites, *μ*m) of basilar and apical dendrites on pyramidal neurons in the three PFC subareas of control and stressed rats. Hemispheric asymmetry refers to the dendritic length in the right compared to the left hemisphere (expressed as percentage). In the PL, chronic restraint stress reduced apical dendritic length exclusively in the right hemisphere (mean ± SEM). *n* is the number of animals from which data were derived.

		Control			Stress		
		
		Total dendritic length (*μ*m)		Hemispheric	Total dendritic length (*μ*m)		Hemispheric
				asymmetry			asymmetry

		L	R	R as % of L	L	R	R as % of L

Basilar dendrites	IL	2928 ± 433	2471 ± 400	84%	2671 ± 441	2530 ± 463	95%
		*n* = 9	*n* = 7		*n* = 9	*n* = 9	
	PL	2779 ± 135	2402 ± 333	86%	2742 ± 416	2257 ± 217	82%
		*n* = 5	*n* = 5		*n* = 11	*n* = 12	
	ACx	3364 ± 406	3220 ± 564	96%	2811 ± 442	2265 ± 281	81%
		*n* = 9	*n* = 9		*n* = 7	*n* = 7	

Apical dendrites	IL	2261 ± 295	2514 ± 383	111%	2204 ± 301	2880 ± 412	131%
		*n* = 9	*n* = 7		*n* = 9	*n* = 9	
	PL	2066 ± 186	3289 ± 625	159%	2397 ± 444	1957 ± 170*	82%
		*n* = 5	*n* = 5		*n* = 11	*n* = 12	
	ACx	2339 ± 212	2618 ± 283	112%	1956 ± 416	2269 ± 408	116%
		*n* = 9	*n* = 9		*n* = 7	*n* = 7	

*P < .05 significant difference to control as determined by two-way ANOVA with Bonferroni's post hoc test. IL, infralimbic cortex; PL, prelimbic cortex; ACx, anterior cingulate cortex; L, left hemisphere; R, right hemisphere.

**Table 2:** Number of branching points and branches in basilar and apical dendrites on pyramidal neurons in PFC subareas.

			Control			Stress		
			
			IL	PL	ACx	IL	PL	ACx

Basilar dendrites	Total number of branching points	L	17.3 ± 2.7 (9)	13.8 ± 0.7 (5)	18.8 ± 2.9 (9)	13.3 ± 1.7 (9)	13.4 ± 1.5 (11)	15.3 ± 2.1 (7)
		R	11.5 ± 1.3 (7)	13.1 ± 1.2 (5)	16.7 ± 2.0 (9)	11.7 ± 1.7 (9)	12.5 ± 1.0 (12)	13.3 ± 2.3 (7)
	Total number of branches	L	40.6 ± 5.6 (9)	35.3 ± 1.7 (5)	44.6 ± 6.2 (9)	32.2 ± 4.1 (9)	30.3 ± 3.7 (11)	37.7 ± 4.1 (7)
		R	30.5 ± 2.6 (7)	32.8 ± 2.7 (5)	41.3 ± 4.2 (9)	30.4 ± 3.9 (9)	31.9 ± 2.2 (12)	32.9 ± 4.9 (7)

Apical dendrites	Total number of branching points	L	12.4 ± 1.5 (9)	11.7 ± 0.8 (5)	13.0 ± 1.5 (9)	11.0 ± 2.3 (9)	11.6 ± 2.0 (11)	11.0 ± 2.3 (7)
		R	15.1 ± 2.4 (7)	17.3 ± 2.9 (5)	14.7 ± 1.6 (9)	13.6 ± 2.9 (9)	**10.5** ± **0.8** ^#^ **(12)**	13.6 ± 2.9 (7)
	Total number of branches	L	25.1 ± 2.7 (9)	24.5 ± 1.8 (5)	27.0 ± 2.9 (9)	23.3 ± 4.8 (9)	25.1 ± 4.1 (11)	23.3 ± 4.8 (7)
		R	31.4 ± 4.9 (7)	36.1 ± 5.9 (5)	30.8 ± 3.2 (9)	28.4 ± 5.8 (9)	22.8 ± 1.7 (12)	28.4 ± 5.8 (7)
	Number of branches (order 3)	L	2.9 ± 0.3 (9)	2.9 ± 0.4 (5)	3.1 ± 0.4 (6)	2.8 ± 0.4 (7)	3.3 ± 0.3 (9)	2.3 ± 0.3 (6)
		R	2.8 ± 0.4 (6)	4.0 ± 0.2 (5)	2.4 ± 0.2 (5)	2.5 ± 0.3 (7)	**2.9** ± **0.3** ^#^ **(8)**	**4.1** ± **0.7** * ^#^ **(6)**
	Number of branches (order 4)	L	2.9 ± 0.3 (9)	2.7 ± 0.3 (5)	3.1 ± 0.4 (6)	2.9 ± 0.4 (7)	3.7 ± 0.6 (9)	2.7 ± 0.4 (6)
		R	**4.3** ± **0.8** * **(6)**	3.1 ± 0.4 (5)	4.0 ± 0.5 (5)	3.5 ± 0.3 (7)	3.3 ± 0.4 (8)	3.7 ± 0.8 (7)
	Number of branches (order 5)	L	3.8 ± 0.3 (9)	2.5 ± 0.4 (5)	3.3 ± 0.3 (6)	3.7 ± 0.6 (7)	4.1 ± 0.8 (8)	3.2 ± 0.7 (5)
		R	3.1 ± 0.4 (6)	3.6 ± 0.2 (5)	3.8 ± 0.5 (5)	3.6 ± 0.5 (7)	3.1 ± 0.3 (8)	3.0 ± 0.4 (7)
	Number of branches (order 6)	L	4.2 ± 0.6 (9)	2.8 ± 0.3 (5)	3.8 ± 0.8 (6)	4.0 ± 0.6 (7)	5.6 ± 1.0 (8)	2.5 ± 0.5 (5)
		R	**2.3** ± **0.3** * **(6)**	4.6 ± 1.2 (5)	4.2 ± 1.2 (5)	3.6 ± 0.3 (7)	3.0 ± 0.3 (8)	3.7 ± 1.0 (7)
	Number of branches (order 11)	L	0 ± 0 (0)	2.2 ± 0.9 (3)	0 ± 0 (0)	0 ± 0 (0)	3.1 ± 0.7 (3)	3.3 ± 0.7 (3)
		R	**6.0** ± **0.0** * **(3)**	2.5 ± 0.8 (3)	2.0 ± 1.1 (3)	3.3 ± 0.8 (3)	3.0 ± 1.0 (3)	**0** ± **0** ^#^ **(0)**
	Number of branches (order 12)	L	0 ± 0 (0)	0 ± 0 (0)	0 ± 0 (0)	0 ± 0 (0)	0 ± 0 (0)	0 ± 0 (0)
		R	**2.7** ± **0.6** ** **(3)**	0 ± 0 (0)	0 ± 0 (0)	**0** ± **0** ^##^ **(0)**	0 ± 0 (0)	0 ± 0 (0)

*P < .05, **P < .01 significant differences between right (R) and left (L); ^#^ *P* < .05, ^##^ *P* < .01 significant difference to *control* as determined by ANOVA with Bonferroni's post hoc test. Numbers in parentheses indicate number of animals from which data (means ± SEM) were derived (n). Abbreviations: IL, infralimbic cortex; PL, prelimbic cortex; ACx, anterior cingulate cortex.
